# Dynamin-related protein Drp1 is required for Bax translocation to mitochondria in response to irradiation-induced apoptosis

**DOI:** 10.18632/oncotarget.4200

**Published:** 2015-06-04

**Authors:** Ping Wang, Peiguo Wang, Becky Liu, Jing Zhao, Qingsong Pang, Samir G. Agrawal, Li Jia, Feng-Ting Liu

**Affiliations:** ^1^ Department of Radiobiology, Key Laboratory of Cancer Prevention and Therapy, National Clinical Research Centre of Cancer, Tianjin Medical University Cancer Institute and Hospital, Tianjin, China; ^2^ East Surrey Hospital, Surrey and Sussex Healthcare NHS Trust, Redhill, Surrey, United Kingdom; ^3^ Pathology Group, Blizard Institute, Queen Mary University of London, London, United Kingdom; ^4^ Centre for Haemato-Oncology, Barts Cancer Institute, Queen Mary University of London, London, United Kingdom

**Keywords:** Bax, DRP1, mitochondrial fragmentation, apoptosis, UV irradiation

## Abstract

Translocation of the pro-apoptotic protein Bax from the cytosol to the mitochondria is a crucial step in DNA damage-mediated apoptosis, and is also found to be involved in mitochondrial fragmentation. Irradiation-induced cytochrome *c* release and apoptosis was associated with Bax activation, but not mitochondrial fragmentation. Both Bax and Drp1 translocated from the cytosol to the mitochondria in response to irradiation. However, Drp1 mitochondrial translocation and oligomerization did not require Bax, and failed to induce apoptosis in Bax deficient diffuse large B-cell lymphoma (DLBCL) cells. Using fluorescent microscopy and the intensity correlation analysis, we demonstrated that Bax and Drp1 were colocalized and the levels of colocalization were increased by UV irradiation. Using co-immuno-precipitation, we confirmed that Bax and Drp1 were binding partners. Irradiation induced a time-associated increase in the interaction between active Bax and Drp1. Knocking down Drp1 using siRNA blocked UV irradiation-mediated Bax mitochondrial translocation. In conclusion, our findings demonstrate for the first time, that Drp1 is required for Bax mitochondrial translocation, but Drp1-induced mitochondrial fragmentation alone is not sufficient to induce apoptosis in DLBCL cells.

## INTRODUCTION

Mitochondrial dynamics is dysregulated in many human diseases including cancer, and it has been recently proposed as a therapeutic target for the treatment of cancer [1–4]. Treatment-induced mitochondrial fission or fragmentation was linked to either apoptotic or necrotic cell death [5, 6]. The dynamin related protein Drp1 - a large GTPase of the dynamin superfamily, is a key protein to induce mitochondrial fission. It is also required to achieve mitochondrial outer membrane permeability (MOMP) with the pro-apoptotic protein Bax, and cytochrome c release from mitochondria [5]. Interestingly, others found that Bax activation is not essential for MOMP, but crucial for Drp1-mediated mitochondrial fission during photodynamic, therapy-mediated apoptosis [7].

Bax is a crucial protein in the activation of both intrinsic and extrinsic apoptotic pathways [8–12]. Bax activation involves translocation from the cytosol to the mitochondria, conformational change, insertion into the mitochondrial outer membrane; oligomerization with itself or other proteins; eventually inducing MOMP and the release of cytochrome *c* from the inter-membrane space [9–11, 13–15]. Although Bax protein levels are crucial for promoting its translocation to the mitochondria [10], the mechanism by which Bax actively moves from the cytosol to the mitochondria is still elusive. It was proposed that Bax cytosolic localization is maintained by interacting with 14–3-3 [16], Ku70 [17] or Parkin [18]. Ultraviolet irradiation resistance-associated gene (UVRAG) inhibits UV irradiation-induced apoptosis by preventing Bax mitochondrial translocation [19]. The BH3-only protein PUMA or Bim can promote Bax mitochondrial translocation by directly interacting with Bax, and indirectly, by competitive binding to Bcl-xL during UV light or TNFα-induced apoptosis [20, 21].

Similar to Bax, Drp1 activation involves mitochondrial translocation, leading to mitochondrial fission or fragmentation. This process requires dephosphorylation of serine 637 of Drp1 (Drp1-P^S637^) [22–24] via activation of the mitochondrial serine/threonine protein phosphatase PGAM5 [6, 25]. However, whether there is clear link between Bax and Drp1 mitochondrial translocation, and whether they are both crucial for the apoptotic cell death is still not clear.

Diffuse large B-cell lymphoma (DLBCL) is the most common and aggressive subtype of non-Hodgkin lymphoma. The cornerstone of treatment is a combination of chemotherapy and immunotherapy, most commonly R-CHOP (rituximab, cyclophosphamide, doxorubicin, vincristine, and prednisone). Efforts to improve upon treatment with R-CHOP have generally proved unsuccessful, and there is a growing body of retrospective and prospective data suggesting a benefit for consolidation radiation therapy in select patients with advanced DLBCL [26, 27]. The levels of Bax expression on treatment response and clinical prognosis on patients with DLBCL are controversial. It was reported that Bax expression is a statistically significant prognostic factor in predicting the overall and disease-free survival of patients with DLBCL [28]. However, others proposed that the expression of Bax and other proteins of the Bcl-2 family have no impact on DLBCL prognosis and treatment response [29, 30].

Previous studies have shown that Bax mitochondrial translocation is a crucial step for UV irradiation-induced apoptosis [13, 19]. In this study, we aimed to determine the role of Bax in UV light-induced mitochondrial fragmentation, and whether Drp1 can promote Bax mitochondrial translocation in a panel of human DLBCL cell lines. The interaction between Bax and Drp1 was determined by both the imaging colocalization analysis, and immuno-precipitation.

## RESULTS

### UV irradiation-induced mitochondrial fragmentation does not require Bax protein

**Figure 1:**
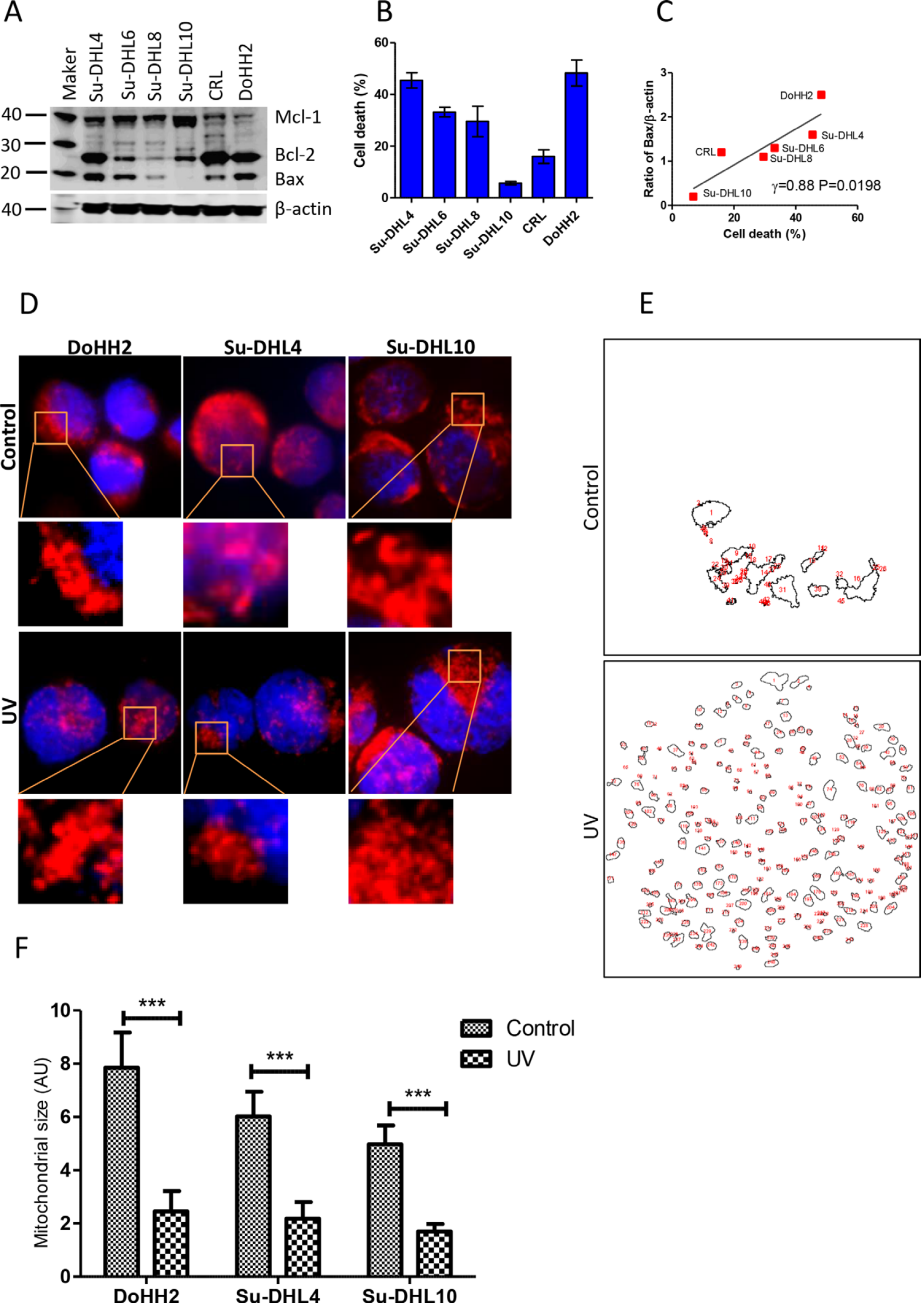
Bax expression and irradiation induced mitochondrial fragmentation **A.** Expression of Bax, Bcl-2 and Mcl-1 in 6 DLBCL cells lines. Mouse anti-Bax (2D2), Bcl-2 (100), and Mcl-1 (B-6) were used for Western blotting. Intensity of each band was determined by densitometry and expressed as a ratio of specific protein to β-actin. **B.** UV light-induced cell death. DLBCL cells (1 × 10^6^ cell/ml) were treated with UV light for 5 min. After 24 hours, cells were stained with PI and died cells (PI+ cells) were determined by flow cytometry. **C.** Correlation between levels of Bax and percentages of cell death. Data were analyzed by Pearson's correlation. Data shown are mean ± SD from three independent experiments. (D-F) UV irradiation-induced mitochondrial fragmentation. DoHH2, Su-DHLDHL4 and Su-DHL10 cell lines were stained with MitoTracker Red and then treated with UV light for 5 min. After 2 hours, cells were co-stained with Hoechst 33342 and then fixed on slides. **D.** Representative cell images showing mitochondrial fragmentation after UV irradiation. Images of mitochondria (red) and nucleus (blue) were collected by the fluorescent microscopy. **E.** Representative mitochondrial outlines from a single cell generated by the ImageJ software. **F.** Statistical analysis of mitochondrial sizes (AU). Three cells with clear mitochondrial outlines were selected and the *Mann*-*Whitney U*-test was used for statistical analysis. AU: Arbitrary unit.

UV irradiation leads to nuclear DNA unwinding in both apoptotic sensitive and resistant cancer cells, but the resistant malignant cells can escape from DNA damage-mediated apoptosis [31]. We tested the association between the expression of Bcl-2 family proteins and the sensitivity to UV irradiation-induced cell death in a panel of DLBCL cell lines, named Su-DHL4, Su-DHL6, Su-DHL8, Su-DHL10, CRL and DoHH2. All cell lines expressed different levels of Bax, Bcl-2 and Mcl-1 and were all found to be Bax positive, with the exception of Su-DHL10 (Figure 1A). DLBCL showed differential sensitivity to UV-induced cell death after treatment for 24 hours. As expected, the Su-DHL10 cell line was highly resistant to UV-induced cell death (Figure 1B). The sensitivities of these cell lines to UV-induced cell death were significantly correlated with the levels of Bax expression (Figure 1C), but no relationship was found with Bak, Bcl-2 or Mcl-1 (Supplementary Figure 1).

The role of mitochondrial fragmentation or fission in cell death is unclear. We observed that UV irradiation-induced mitochondrial fragmentation could be seen from as early as 2 hours after treatment. Mitochondrial fragmentation occurred in both the Bax positive DoHH2/Su-DHL4 cell lines, and the Bax negative Su-DHL10 cell line (Figure 1D and 1E). Cell death was not detected at this time point. Significantly reduced mitochondrial sizes were detected in these three cell lines after UV irradiation; regardless of Bax expression (Figure 1F). These results indicate that UV irradiation-induced mitochondrial fragmentation is Bax-independent. However, mitochondrial fragmentation alone is not sufficient to induce cell death in Bax negative cells.

### Irradiation-induced p-Drp1-(S637) dephosphorylation is correlated with PGAM5 activation

**Figure 2:**
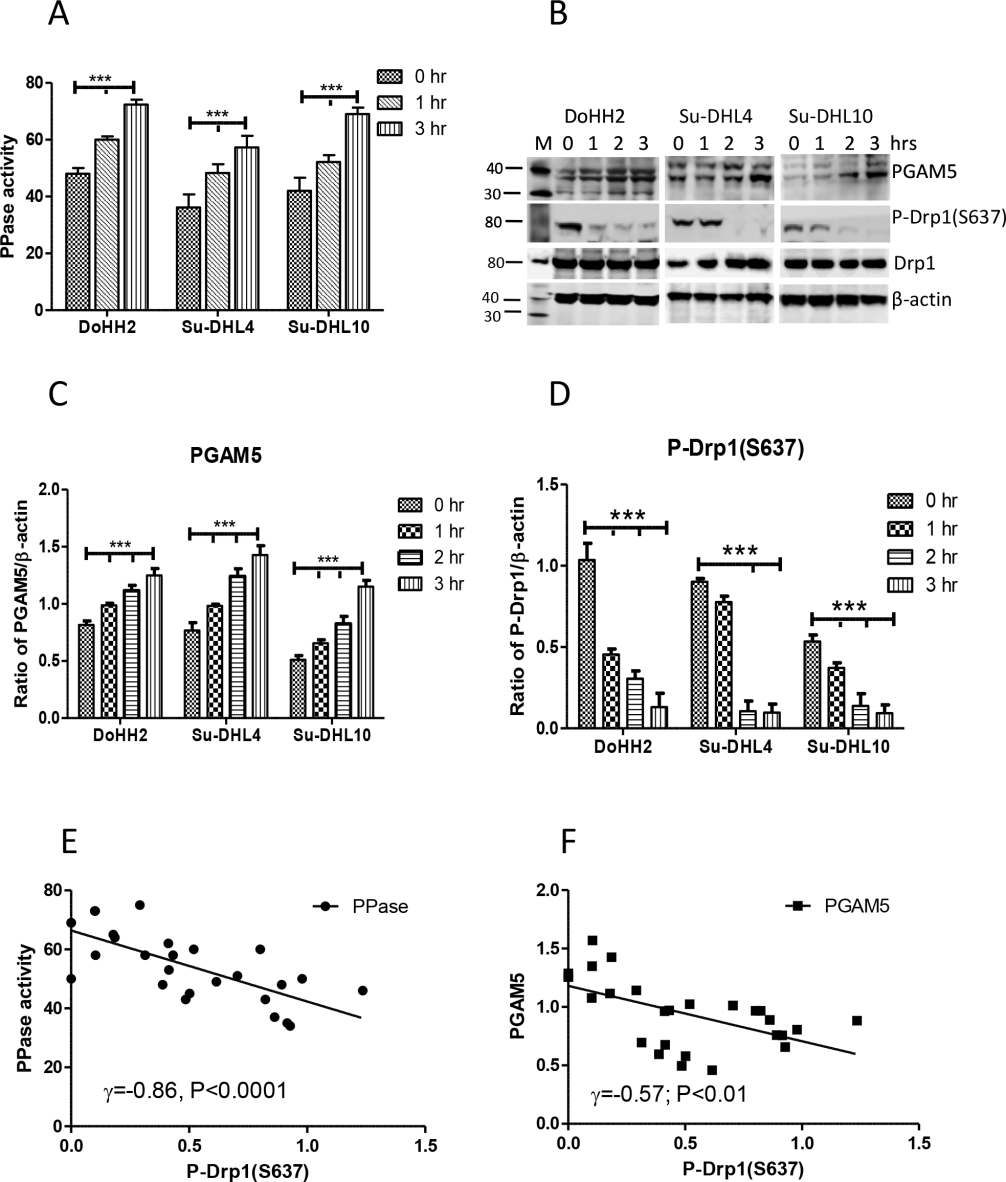
The association between PGAM5 activation and DRP1dephosphorylation **A.** Activation of PPase. Proteins in 10 μg/10 μl were used for the enzyme assay. **B.** Expression of PGAM and p-DRP1-(S637). Polyclonal goat anti-PGAM5 antibody was used at 1:200 dilution and rabbit anti-p-DRP1-(S637) antibody was used at 1:1000 dilution. “M” indicates marker for protein molecular weight. **C** and **D.** Statistical analysis of the expression levels of PGAM5 (C), or p-DRP1-(S637) (D). Ratios of PGAM5 or p-DRP1-(S637) to β-actin (mean ± SD) were determined by densitometry from 3 independent experiments and statistical analysis was performed by two-way ANOVA. **E** and **F.** Correlation between PPase activity (E) or PGAM5 (F) with p-DRP1-(S637). Pearson's correlation was used for statistical analysis.

Mitochondrial fission is associated with Drp1 mitochondria translocation, after the dephosphorylation of its serine 637 site by the mitochondrial protein phosphatase (PPase) PGAM5 [6, 24, 32]. UV irradiation significantly increased both PPase activity and the levels of PGAM5 protein (Figure 2A, 2B, and 2C). This was accompanied by a significantly decreased phosphorylation of p-Drp1-(S637) (Figure 2B and 2D). The decreased phosphorylation of p-Drp1-(S637) was significantly and negatively correlated with the increased PPase activity and the levels of PGAM5 (Figure 2E and 2F), suggesting that p-Drp1-(S637) dephosphorylation was mainly induced by the activation of PGAM5 in response to UV irradiation.

### UV irradiation induces Bax-independent Drp1 oligomerization and mitochondrial translocation

**Figure 3:**
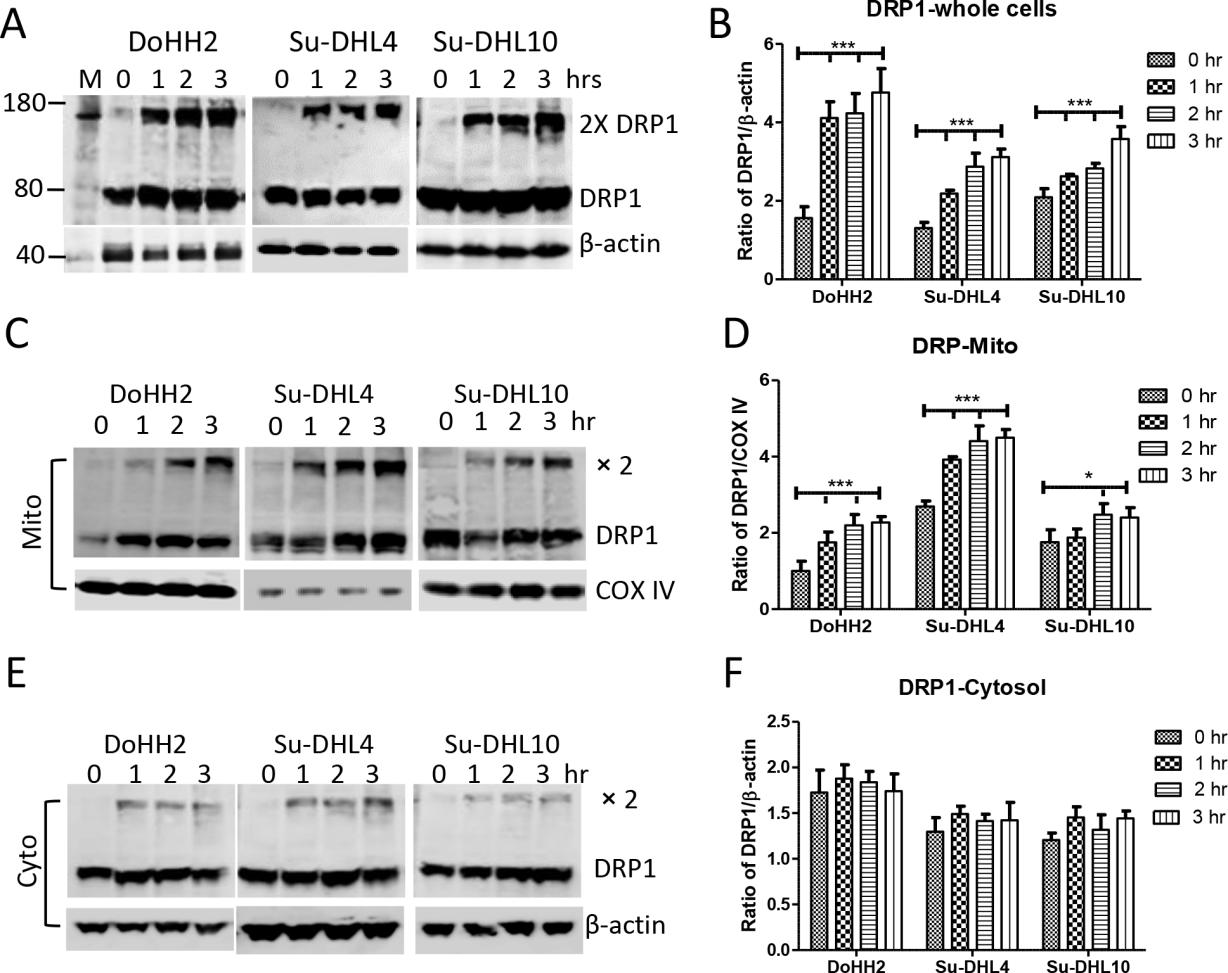
Irradiation induced DRP1 mitochondrial translocation and oligomerization Cells were treated with UV light for 5 min and collected for protein extraction at each indicated time point. UV light-induced changes in DRP1 expression in whole cells **A** and **B**. mitochondrial **C** and **D.** and cytosolic **E** and **F.** fractions. (A, C and E) Representative Western blotting. Monoclonal anti-DRP1 antibody was used at 1:1000 dilution. Monoclonal mouse anti-β-actin (Sigma) and anti-COX IV antibodies were used as loading control for cytosolic and mitochondrial proteins, respectively. (B, D and F) Statistical analysis of UV-light-induced increase in DRP1 expression. Protein expression was presented as ratio of DRP1 (monomer plus dimers) to β-actin or COX IV. Data shown were from 3 independent experiments and statistical analysis was performed by two-way ANOVA.

Mitochondrial fission requires the action of Drp1 mitochondrial translocation [33]. We observed that UV light irradiation induced Drp1 dimerization, and significantly increased the expression of Drp1 in both the Bax positive DoHH2/Su-DHL4 and the Bax negative Su-DHL10 cell lines (Figure 3A and 3B). Using differential detergent fractionation (DDF) technique, we found that significantly increased levels of Drp1 expression and oligomerization were mainly shown in the mitochondrial fraction (Figure 3C and 3D), but not in the cytosolic fraction (Figure 3E and 3F). The significantly increased Drp1 expression was detected from as early as one hour after UV irradiation.

**Figure 4:**
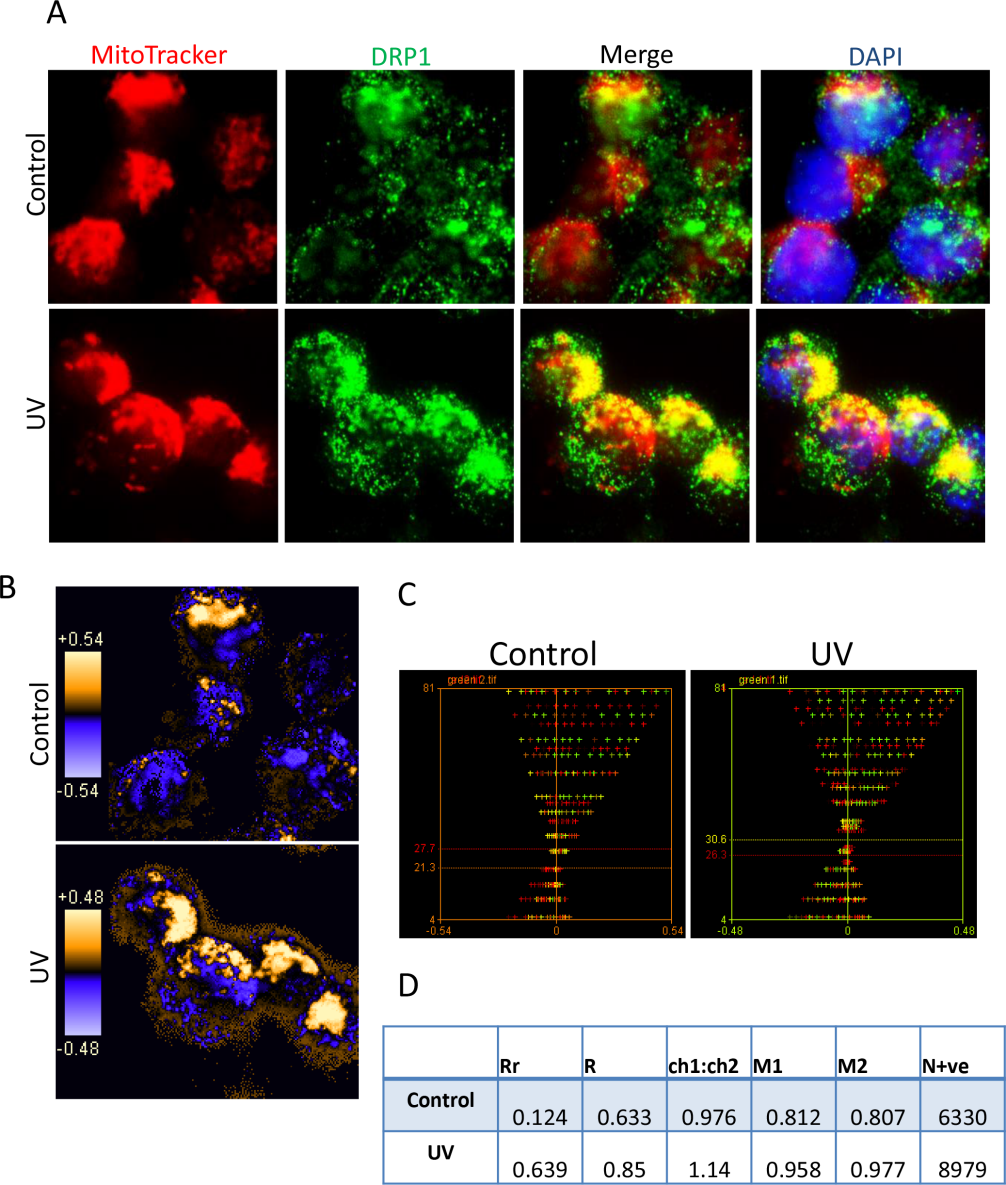
Colocalization analysis of DRP1 with mitochondria **A.** Representative images for immuno-fluorescent staining of DRP1 and mitochondria. Su-DHL4 cells were stained with 50 nM MitoTracker Red and then treated with UV light. After 2 hours' cell culture period, cells were washed, fixed/permeabilized on slides and stained with primary anti-DRP1 antibody (1:20 dilution)/secondary AF-488 conjugated anti-mouse IgG antibody (1:100 dilution). Nucleus was stained with DAPI. Yellow dots indicate DRP1 (green) on the mitochondria (red). **B.** Qualitative analysis of colocalization by PDM images. The PDM image is pseudo-colored, generated by pixel that is equal to the PDM value at that location. The orange color indicates colocalized pixels and the blue color means segregation. **C.** Intensity correlation analysis. The intensity of red or green color was first analyzed individually to obtain red or green ICD plots. Then red and green ICD plots were merged to generate a combined ICD plot. Red and green lines indicate intensities of each color. **D.** Statistical data of colocalization. Data were analyzed by intensity correlation analysis as described in the Materials and Methods.

We performed fluorescent microscopy and the imaging colocalization analysis to confirm UV irradiation-induced Drp1 mitochondrial translocation. The Bax positive Su-DHL4 (Figure 4), and the Bax negative Su-DHL10 (Supplementary Figure 2) cell lines were stained with MitoTracker Red and the DRP1 antibody (showing green color). In untreated cells, Drp1 showed mainly green pixels, indicating the cytoplasmic localization, but partially colocalized with mitochondria, showing yellow pixels. After 2 hours of treatment, the image displayed more yellow pixels, suggesting an increased colocalization of Drp1 with mitochondria (Figure 4A and Supplementary Figure 2A). The qualitative analysis (using PDM images) showed that overlapped pixels (the orange pixels) and segregation (the blue pixels) appeared in both the control and the UV treated cells. As expected, the UV-treated cells showed greater levels of colocalization of Drp1 with mitochondria (Figure 4B and Supplementary Figure 2B). The quantitative analysis was conducted using the intensity correlation analysis (ICA). The red (mitochondria) and the green (Drp1) colors were designated as ‘channel 1’ and ‘channel 2’, respectively. ICA plots of channel 1 (CH1) and channel 2 (CH2) were first generated separately (Supplementary Figure 3), and then merged as a single ICA plot. In Su-DHL4 cells, the difference of intensities in the control was 6.4 [27.7 (red)-21.3 (green)], and decreased to 4.3 [30.6 (green)-26.3 (red)] after irradiation, indicating an increased colocalization (Figure 4C). Similarly, the difference of intensities decreased from 17.5 [30.6 (red)-12.5 (green)] in the control to 0.8 [29.0 (green)-28.2 (red)] in Su-DHL10 cells (Supplementary Figure 2C). Statistical analysis data demonstrated that UV irradiation promoted Drp1 mitochondrial translocation by increasing the Pearson's correlation coefficient (Rr) from 0.124 to 0.639 in the Su-DHL4 cell line, and from 0.339 to 0.438 in the Su-DHL10 cell line. The overlap coefficient (R) and the number of pixel pairs that have a positive PDM value (N+ve) were all increased in UV irradiated Su-DHL4 and Su-DHL10 cells, compared with their controls (Figure 4D and Supplementary Figure 2D). These results further confirmed that UV irradiation induces Drp1 mitochondrial translocation, regardless of Bax expression.

### UV irradiation-induced apoptotic cell death is Bax-dependent

**Figure 5:**
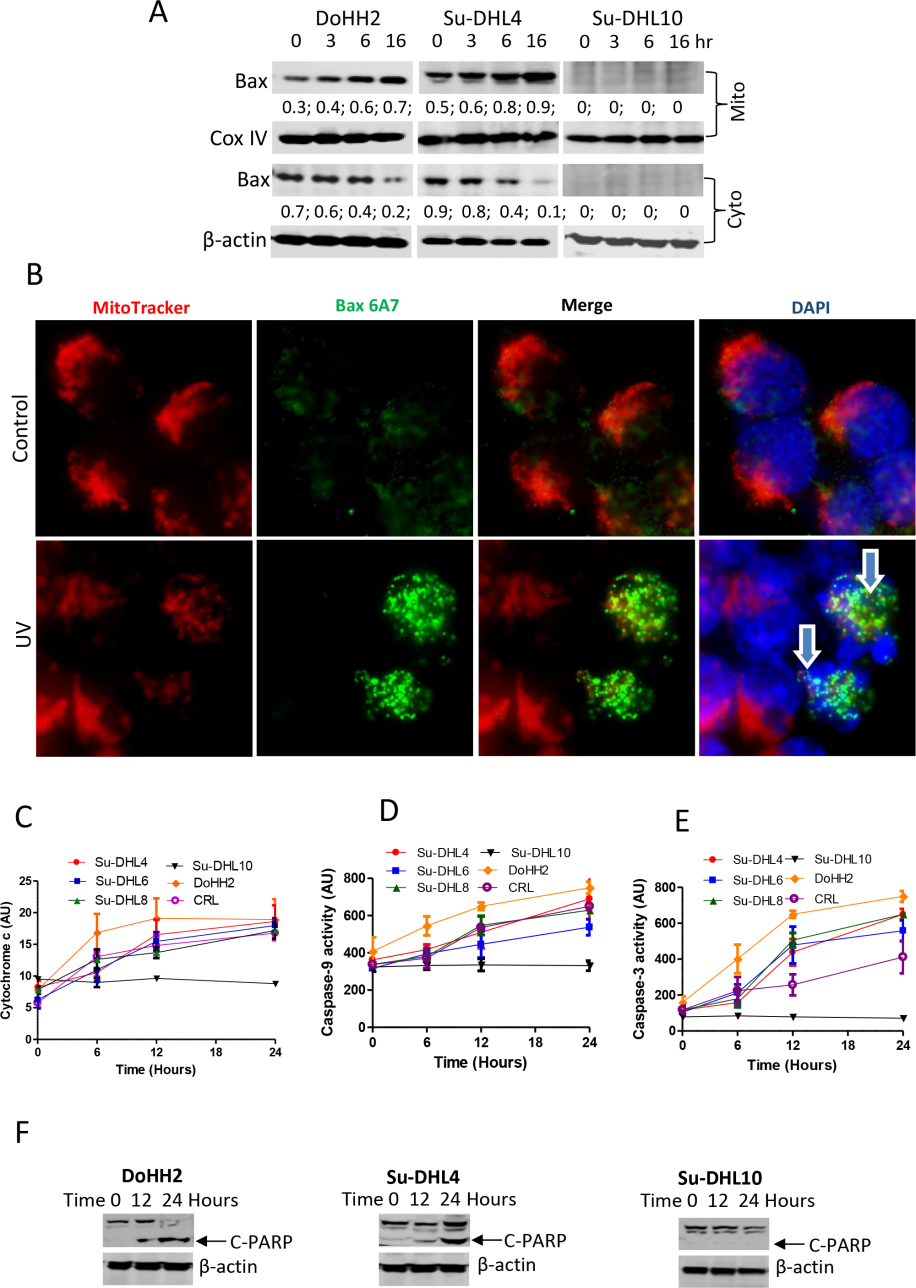
UV irradiation induced Bax activation and apoptosis **A.** Bax translocation to mitochondria. After UV irradiation, mitochondrial and cytosolic fractions were prepared by DDF method and proteins from each fraction were extracted individually for Western blotting. Anti-Bax 2D2 antibody (Santa Cruz) was used at 1:1000 dilution. ‘Mito’ and ‘Cyto’ mean mitochondrial and cytosolic fractions, respectively. Numbers under the Bax blot are ratios of Bax to COX IV or β-actin. **B.** Bax activation. MitoTracker Red pre-stained Su-DHL4 cells were irradiated with UV light and incubated for 4 hours. Cells were washed, fix/permeabilized on the slide and then stained with primary anti-Bax 6A7 antibody (1:100 dilution)/secondary AF-488 conjugated anti-mouse IgG antibody (1:100 dilution). Yellow dots indicate active Bax (green) on the mitochondria (red) location. Arrows indicate apoptotic cells with fragmented nucleus. Proteins from the cytosolic fraction were also used to determine cytochrome *c* release **C.** activation of caspase-9 **D.** and caspase-3 **E.** Data shown are mean ± SD from 3 independent experiments. **F.** UV light-induced PARP cleavage. Cell lysates were extracted from whole cells at each indicated time points. Rabbit anti-PARP antibody was used for detecting cleaved PARP (C-PARP) at 89 kD.

Although Drp1 mitochondria translocation was detected in all the DLBCL cell lines tested, UV irradiation-induced Bax translocation from cytoplasm to mitochondria was only detected in the Bax-positive DoHH2 and Su-DHL4 cell lines (Figure 5A). UV irradiation-induced Bax activation in the Su-DHL4 cell line was confirmed by fluorescent microscopy using the anti-Bax 6A7 antibody, which only detects active Bax (Figure 5B). UV light-induced cytochrome *c* release (Figure 5C), activation of caspase-9 and caspase-3 (Figure 5D and 5E), and PARP cleavage (Figure 5F) were only detected in the Bax-positive cell lines. UV irradiation did not induce Bax expression and downstream apoptotic events in the Bax negative Su-DHL10 cell line. These results suggest that Bax translocation to mitochondria is crucial for UV irradiation-induced apoptosis, but the role of Drp1 was less significant.

### Drp1 is required for Bax mitochondrial translocation

**Figure 6:**
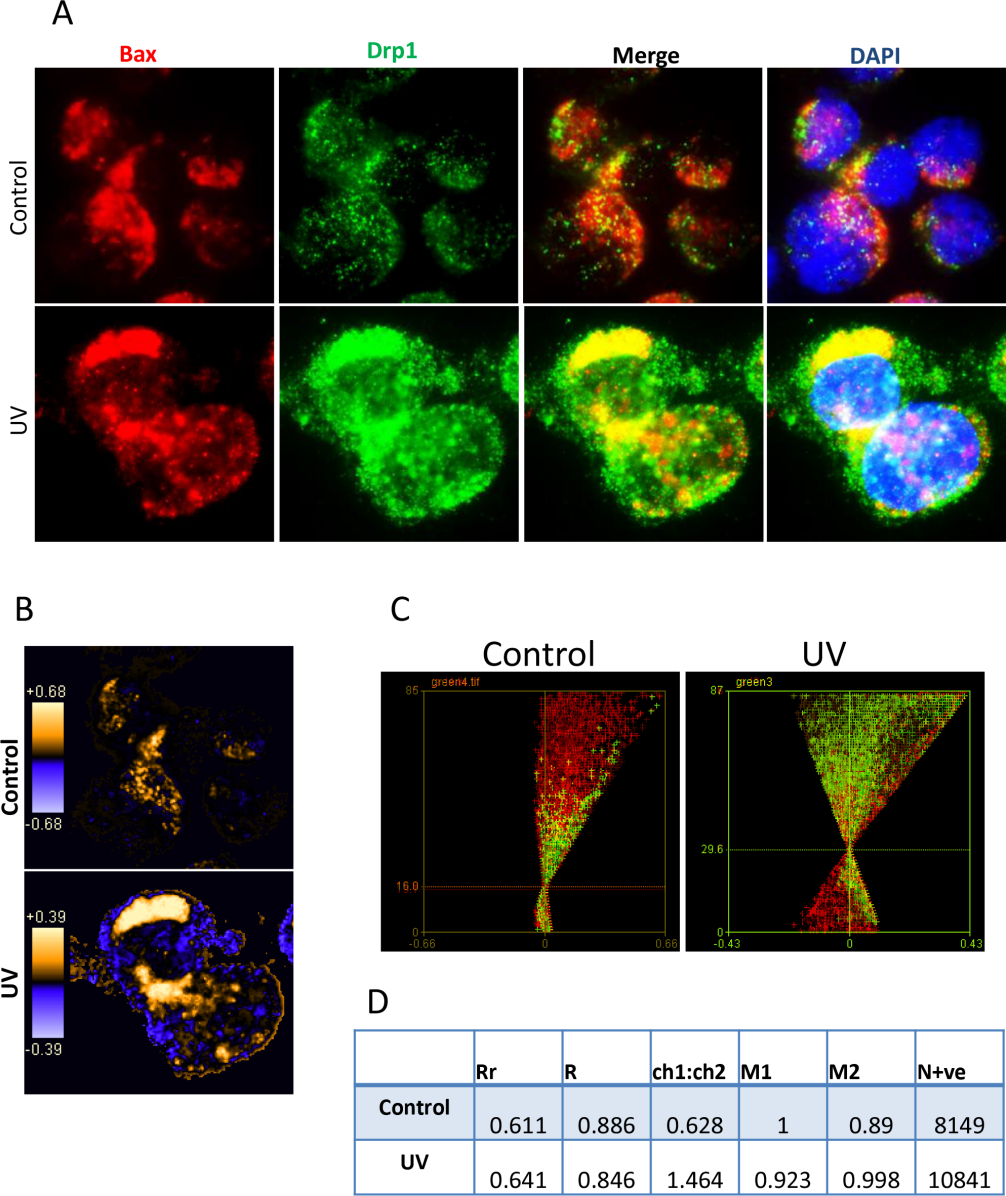
Imaging analysis of Bax and Drp1 colocalization **A.** Immuno-staining of Bax and Drp1. Su-DHL4 cells was treated with UV light for 5 min. After incubation for 2 hours, control and UV-treated cells were fix/permeabilized on slides and co-stained with rabbit anti-Bax (N20) and mouse anti-Drp1 antibodies. AF-546 goat anti-rabbit IgG (red) was used to probe Bax and AF-488 donkey anti-mouse IgG (green) was employed to probe Drp1. Yellow pixels indicate colocalization. **B.** PDM images for Bax and Drp1 colocalization. Orange or blue color pixels indicate colocalization or segregation, respectively. **C.** ICA plots. Combined ICA plots were generated by merging the channel 1 and the channel 2 ICA plots. **D.** Statistical data of colocalization. Data were analyzed by imaging correlation analysis using ImageJ software.

The mechanisms by which Bax and Drp1 translocate to mitochondria are unclear. We were interested in whether Bax and Drp1 translocate to mitochondria simultaneously as binding partners. We first used fluorescent microscopy and imaging analysis to determine whether Bax and Drp1 share same localization before and after irradiation. Bax (N-20) antibody which detects the pan Bax protein was used for co-staining with the Drp1 antibody on Bax positive Su-DHL4 cells. We found that Bax (red) and Drp1 (green) showed partial colocalization (yellow) in both the control and the UV-treated cells (Figure 6A). Bax and Drp1 colocalization was confirmed qualitatively using PDM images. Orange pixels indicate colocalization, and blue pixels represent segregation (Figure 6B). To quantify Bax and Drp1 colocalization, Bax and Drp1 were designated as ‘channel 1′ and ‘channel 2′, respectively. ICA plots were first generated individually (Supplementary Figure 4) and merged together as a combined plot (Figure 6C). The intensities between Bax and Drp1 were similar in both the control and after irradiation. However, both the intensity levels increased from 16.0 in the control, to 29.6 after UV irradiation for 2 hours. ICA statistical data showed that the Rr values were only slightly increased from 0.611 in the control, to 0.641 after treatment, indicating that Bax and Drp1 interaction could be independent of apoptotic signal. However, the N+ve values increased from 8149 in the control to 10841 in UV-treated cells (Figure 6D), suggesting that UV irradiation promotes Bax and Drp1 interaction.

**Figure 7:**
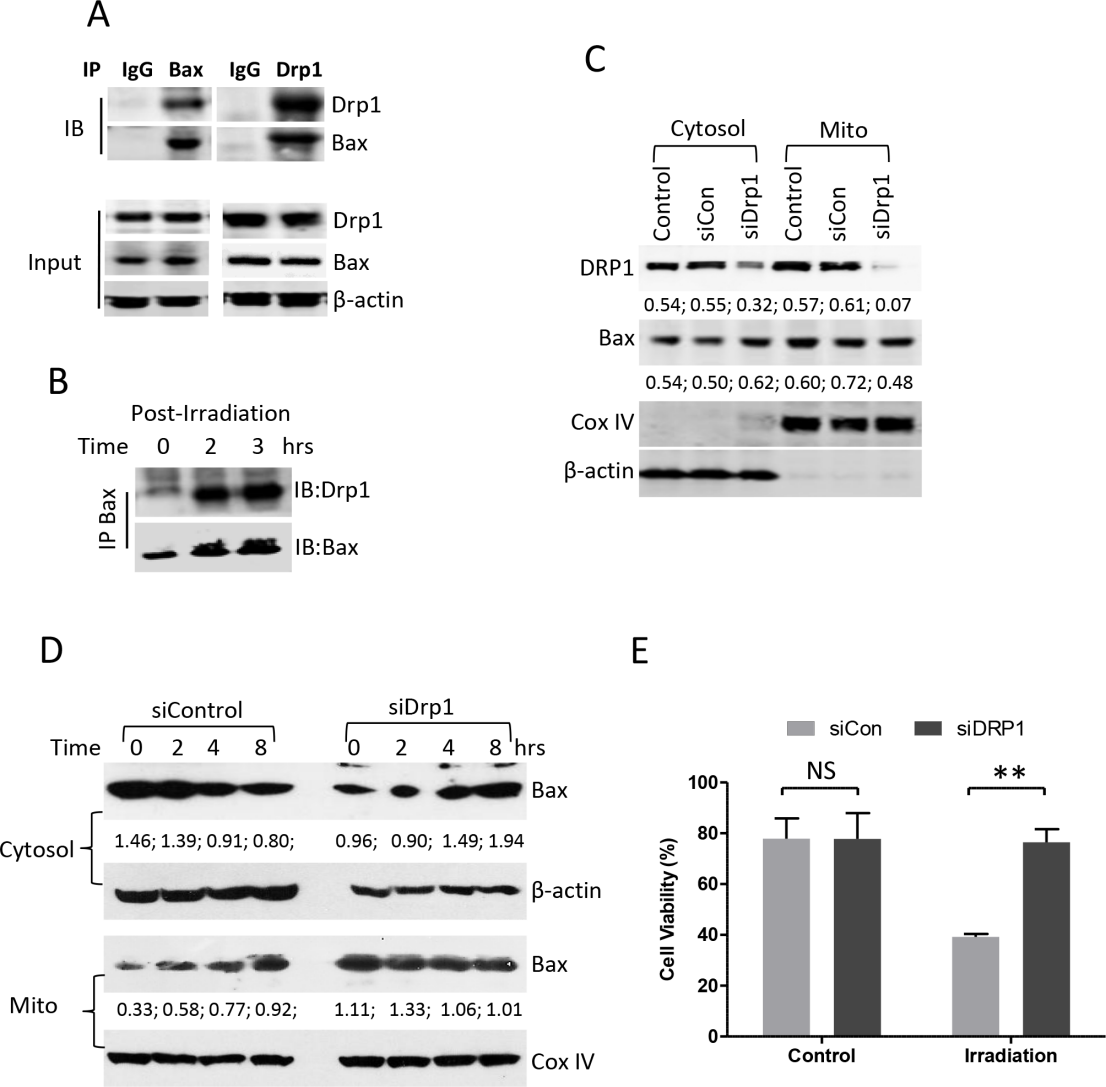
Effect of Drp1 on Bax mitochondrial translocation **A.** Interaction between Bax and Drp1 in the resting stage. Proteins were extracted from untreated Su-DHL4 cells. 500 μg proteins, 5 μg of control mouse IgG, rabbit anti-Bax (N-20) and rabbit anti-Drp1 (H-300) antibodies were used for co-IP and mouse anti-Bax (2D2), or mouse anti-Drp1 (6Z-82) antibodies were used for Western blotting (IB), respectively. **B.** Interaction between Bax and Drp1 after irradiation. Su-DHL4 cells were treated with UV light for 5 min and proteins were extracted using 1% CHAPS-containing buffer after incubation for 2 and 3 hours. Bax (6A7) antibody was used for IP and rabbit anti-Drp1 (H-300) and rabbit anti-Bax (N-20) antibodies were used for Western blotting. (C and D) Knocking-down Drp1. Su-DHL4 cells were transfected with control siRNA (siCon) or Drp1 siRNA (siDrp1) for 24 hours. **C.** Expression of Drp1 and Bax in cytosolic and mitochondrial fractions were determined by Western blotting using rabbit anti-Drp1 and anti-Bax (2D2) antibodies. **D.** After knocking down Drp1 for 24 hours, cells were treated with UV light. Bax expression was determined in both cytosolic and mitochondrial fractions using mouse anti-Bax (2D2) antibody. COX IV and β-actin antibodies were used as loading controls for mitochondria and cytosol, respectively. Numbers under each plot are ratios of specific proteins to loading controls. **E.** After transfection with control siRNA (siCon) or Drp1 siRNA (siDrp1) for 24 hours, DoHH2 cells were treated with UV light for 6 hours. Cell viability was determined by ViCELL-XR Cell Viability Analyzer. Data shown are mean ± SD from 3 independent experiments.

We next determined whether Bax and Drp1 directly bind to each other in the resting stage and in response to the treatment. Bax-Drp1 interaction was detected by Co-IP using either pan Bax (N-20) or Drp1 antibody in the control Su-DHL4 cells (Figure 7A). Increased levels of active Bax and Drp1 binding were also detected after UV irradiation (Figure 7B). To determine whether Drp1 is essential for Bax translocation, Drp1 protein was partially knocked down by transfection with siRNA-Drp1. After transfection for 24 hours, reduced Drp1 protein expression was observed in both cytosolic and mitochondrial fractions. Bax levels were not affected in the cytosol, but were decreased in the mitochondrial fraction after knocking down Drp1 for 24 hours (Figure 7C). In response to UV irradiation, Bax levels were decreased in the cytosolic fraction, and were increased in the mitochondrial fraction of the Su-DHL4 cells transfected with control siRNA, indicating that Bax mitochondrial translocation occurred. Importantly, Bax in the Drp1-deficient cells was accumulated in the cytosolic fraction and the levels of mitochondrial Bax remained unchanged, suggesting that Bax translocation to mitochondria was blocked after UV irradiation (Figure 7D). As a consequence, UV irradiation-induced cell death and caspase-3 activation were significantly reduced in Drp1-deficient cells (Figure 7E and Supplementary Figure 5). These results demonstrate that Bax may rely on Drp1 for its translocation to mitochondria when cells are undergoing apoptosis.

## DISCUSSION

In this article, we report for the first time, that Bax and Drp1 are binding partners and Drp1 promotes Bax mitochondrial translocation. Drp1 mitochondrial translocation and mitochondrial fragmentation alone are not sufficient to induce apoptosis in a cell which lacks Bax expression. Bax activation is crucial for UV irradiation-induced mitochondria-dependent apoptosis, but is not required for mitochondrial fragmentation.

Bax mitochondrial translocation is a crucial step for inducing apoptotic cell death via the intrinsic apoptotic pathway [10, 34, 35], and Drp1 mitochondrial translocation is essential for mitochondrial fission or fragmentation [36, 37]. The association between Bax and Drp1 has previously been reported at the mitochondrial levels. Both Bax and Drp1 translocate to discrete foci on the mitochondria, where mitochondrial Bax stabilizes Drp1, suggesting that Bax participates in apoptotic fragmentation of mitochondria [37–39]. Mitochondrial Drp1 promotes Bax oligomerization, and leads to cytochrome *c* release from mitochondria [5]. Conversely, others have reported that Drp1 is dispensable for apoptotic cytochrome *c* release in response to the Bcl-2 antagonist, ABT-737 [40]. Moreover, it was found that Mcl-1 inhibitors, BI97C1 (Sabutoclax) and BI112D1 induced mitochondrial fragmentation, which is independent of Drp1, and is upstream or independent of apoptosis [41].

We found that UV irradiation induced Drp1 translocation and mitochondrial fragmentation in both Bax-positive DoHH2/Su-DHL4 cell lines, and Bax-negative Su-DHL10 cells. The Su-DHL10 cell line is not only Bax negative, but also lacks expression of Bak protein, whereas DoHH2 and Su-DHL4 cells are Bak expressing cells (Supplementary Figure 1A). The sensitivities of DLBCL cells to UV irradiation-induced cell death were also positively correlated with the levels of Bak, but the correlation was not significant (Supplementary Figure 1B). Our main focus in this study was on the mechanism of Bax mitochondrial translocation, but Bak protein is a mitochondrial protein. We do not exclude the pro-apoptotic role of Bak in UV irradiation-induced apoptosis whilst focusing on Bax. UV irradiation-induced cytochrome *c* release and activation of caspases only occurred in the Bax-positive cells. This suggests that Drp1-mediated mitochondrial fragmentation is Bax-independent, at least in these DLBCL cell lines. Interestingly, UV-induced mitochondrial fragmentation alone failed to induce cytochrome *c* release and apoptosis in Bax-negative cells, indicating that mitochondrial fragmentation is not a cause for MOMP and cytochrome *c* release, therefore it is not crucial for apoptotic cell death. About 20% dead cells were detected in Su-DHL10 cells after UV irradiation for 72 hours, suggesting that Su-DHL10 cells either died of necrosis, or a caspase-independent cell death. It has been well-evidenced that apoptosis-resistant cancer cells die slowly in response to DNA damage when apoptosis downstream events are blocked by expression of oncogenic Ras, Raf, mitogen-activated kinases and apoptosis inhibitor Smac/Diablo [42–45], or deficiency of Apaf-1 [31].

One of our main aims of this study was to determine the interaction between Drp1 and Bax before and after treatment. Using fluorescent microscopy and imaging colocalization analysis, we identified by both qualitative and quantitative methods that Bax and Drp1 shared the same location in the resting DLBCL cells. Co-IP result confirmed that pan-Bax and Drp1 interacted with each other in untreated cells. These results strongly suggest that pan-Bax and Drp1 are binding partners before their mitochondrial translocation. We tempted to determine interaction between Bax and p-Drp1-(S637), but the outcomes were not satisfactory enough to show any positive result. Therefore, we are not able to conclude whether Bax interacts with p-Drp1-(S637) or its non-phosphorylated form in the cytosol. In response to UV irradiation, both pan-Bax and Drp1 translocated to the mitochondria. Increased colocalization of pan-Bax and Drp1 were indicated by increased PDM, and N+ve values compared with the data of untreated control. Using anti-active Bax antibody, we detected that UV irradiation increased the interaction between the conformational changed Bax and Drp1 in a time-dependent manner. This suggests that the interaction between Bax and Drp1 at the mitochondrial levels may be associated with the apoptotic process.

The DLBCL Su-DHL4 cell line expressed certain levels of Bax in both the cytosol and the mitochondria. This phenomenon was also previously observed in other DLBCL cell lines, CRL and DoHH2 [11]. The pro-apoptotic effect of mitochondrial Bax was overwhelmed by overexpression of Bcl-2 in these cells. The sensitivity of cancer cells to an apoptotic stimulus is dependent on the ratio of Bax/Bcl-2 or Bcl-xL at the mitochondrial levels [9, 10, 13, 43]. Therefore, Bax mitochondrial translocation is an important step to increase the sensitivity of cancer cells to treatment-induced apoptosis.

It is obvious that Drp1 mitochondrial translocation and oligomerization can be Bax-independent because it had occurred in the Bax-negative cells. This observation was consistent with a previous report which stated that mitochondrial fragmentation could be either upstream of independent of apoptosis [41]. Interestingly, Bax mitochondrial translocation was blocked in the Drp1 knocking-down cells after treatment with UV. Increased Bax expression was accumulated in the cytosol, rather than at the mitochondrial levels. This result proposes that Bax mitochondrial translocation requires the assistance of Drp1.

Unlike the pro-apoptotic protein Bax, the role of Drp1 in cancer development and treatment is complex. Up-regulated expression of Drp1 and increased mitochondrial fission were found in human lung cancer cells [46]. Inhibition of Drp-1-mediated mitochondrial fission by mdivi-1 prevented cell cycle progression, induced apoptotic cell death in lung and colon cancers [46–48] and enhanced the efficacy of chemotherapy by platinum [49]. Therefore, it has been suggested that Drp1 could be a therapeutic target for the anti-cancer therapy [50]. However, our study demonstrates that Drp1 promotes Bax translocation and apoptosis in response to UV irradiation.

In summary, we verified the interaction between Drp1 and Bax in human DLBCL cell lines using both imaging colocalization analysis and immuno-precipitation. Drp1 alone does not have pro-apoptotic role but it promotes Bax mitochondrial translocation in response to UV irradiation. However, Drp1 expression, its roles in radio- or chemo-therapy, and its prognostic values need to be investigated in primary DLBCL samples.

## MATERIALS AND METHODS

### Cell lines, cell culture and UV light irradiation

DLBCL cell lines DoHH2, CRL [11], Su-DHL4, Su-DHL6, Su-DHL8 and Su-DHL10 [51–53] were used in this study. Cells were cultured in RPMI-1640 medium supplemented with 10% heat-inactivated fetal calf serum (FCS), 25 mM HEPES, and 2.0 mM L-glutamine at 37°C in a 5% CO_2_ humidified incubator. DLBCL cells (1 × 10^6^/ml) were exposed to UV irradiation (120 mJ/cm2) (model TM-20; Chromato-UV-E Transilluminator) for 5 min and then cultured for further experiment [31].

### Determination of cell death by flow cytometry

Cells were incubated with 50 μg/ml PI (propidium iodide, Sigma) and the fluorescence of PI was measured on the FL3-H channel by FACS flow cytometry (BD) [11].

### Ser/Thr ppase assay

Ser/Thr PPase activity was determined with a RediPlate 96 EnzChek tyrosine phosphatase assay kit (Life Technologies) in accordance with the manufacturer's recommendations. In brief, cells were suspended in fluorogenic assay buffer (20 mM HEPES-KOH, pH 7.4, 10 mM DTT, 10% sucrose, 1.0 mM EDTA, 0.1% CHAPS). After centrifugation at 12, 000 × *g* for 15 min, 50 μl of supernatant, containing 50 μl of proteins, was placed into RediPlate wells and incubated for 25 min at 22°C before reading for fluorescence. DiFMU was determined at 380/460 nm using a BMG LABTECH POLARstar OPTIMA Microplate Reader (Offenburg, Germany).

### Immuno-staining and fluorescent microscopy

For determine mitochondrial fission, cells were stained with 50 nM MitoTracker Red CMXRos (Life Technologies) and then irradiated by UV light. For immuno-staining, cells on slides were fixed and permeabilized with Cytofix/Cytoperm reagents (BD) and blocked with a buffer consisting of 0.1% saponin and 5% serum (the type of serum corresponding to the isotype of the secondary antibody). Cells were stained with monoclonal Drp1, or monoclonal Bax clone 6A7, or co-stained with monoclonal Drp1 and polyclonal Bax N20 antibodies for 1 hour at room temperature. After washing with TBST (TBS containing 0.1% Tween-20), cells were incubated with Alexa-Fluor conjugated secondary antibodies at 1:100 dilution. Details of antibodies used for this experiment were listed in the Supplementary Tables 1 and 2. Slides were washed for 3 times with TBST, stained with 50 ng/ml DAPI (4′,6-diamidino-2-phenylindole, Sigma), air-dried at 4°C in the dark, and mounted in ProLong^®^ Gold anti-fade reagent (Life Technologies) before being viewed under an Olympus BX40 fluorescence microscope (Artisan Scientific Corporation) [54].

### Image analysis

Mitochondrial fragmentation and Drp1 colocalization with mitochondria or Bax were determined by particle and colocalization analysis, respectively, using WCIF ImageJ software (Wright Cell Imaging Facility). The size of the DLBCL cell was set up as 40 arbitrary (AU) units for the measurement scales. Before the analysis, images were converted to 8-bit grayscales. The particle analysis method was used to analyze the sizes of mitochondria. A “threshold” range was set and pixels in the image whose value lay in this range were converted to black; pixels with values outside this range were converted to white. The particles with clear outlines were counted as mitochondria.

Colocalization analysis was based on the theory that if two proteins are parts of the same complex, then their staining intensities should vary in synchrony, whereas if they are on different complexes or structures, they will exhibit asynchronous staining. Before analysis, the red and green images were converted to 8-bit grayscale pictures and the intensive correlation analysis (ICA) method was used for determine the levels of colocalization [55]. Image of (PDM): the ***P***roduct of the ***D***ifferences from the ***M***ean, i.e. for each pixel: (red intensity ─ mean red intensity) × (green intensity – mean green intensity) was used to qualitative analysis of colocalization. ICA describes the intensity synchrony between channels 1 and 2 [56]. The axes on the ICA plots are the PDM values on the x-axis and the red or green intensity on the y-axis. A combined ICA plot was generated by merging the red and the green ICA plots to quantitative analysis of the levels of colocalization. Statistical data for the colocalization levels, Pearson's correlation coefficient (Rr), overlap coefficient (R), red:green pixel ratio (Ch1:Ch2), colocalization coefficients for channel 1 (M1) and channel 2 (M2), and the number of pixel pairs that have a positive PDM value (N+ve), were generated by the intensive correlation analysis program (http://www.uhnresearch.ca/facilities/wcif/imagej).

### Differential detergent fractionation for cellular proteins

Differential detergent fractionation (DDF) involves sequential extraction of cells using detergent-containing buffer, separating cellular proteins into distinct compartments [57, 58]. The basic buffer for this experiment contains 10 mM PIPES, pH6.8, 300 mM sucrose, 100 mM NaCl, 3 mM MgCl_2_, 5 mM EDTA and protease inhibitor cocktails (Sigma). To extract cytosolic fraction (fraction 1), DLBCL cells were suspended into the buffer 1 which contains 0.02% digitonin, which only permeabilizes the plasma membrane and incubated on ice for 10 min. After centrifugation at 8000 rpm for 2 min, the cytosolic extracts were transferred into Eppendorf tubes. The pellets were washed with buffer 1 to reduce cytosolic protein contamination. Fraction 2 proteins (mitochondria and membranous organelles) were extracted with the buffer 2 containing 0.5% Triton X-100. Identification of cellular protein fractions were performed by Western blotting using specific antibodies: β-actin for cytosolic and COX IV for mitochondrial proteins, respectively.

### Western blotting

Proteins were then extracted with CelLytic^™^ M cell Lysis Reagent (Sigma) supplied with protease inhibitor cocktails. Protein concentration was determined by Bradford method, using Bio-Rad Bradford reagent. The proteins were mixed with NuPAGE LDS Sample Buffer and boiled for 5 min before analysis by Western blotting. Proteins were subjected to 4–12% NuPAGE gels (Life Technologies), and transferred onto PVDF membrane (Sigma) at 20 V for 1 hour by semi-dry transfer. PVDF membrane was blocked with blocking buffer (5% polyvinyl pyrrolidone, 5% FCS and 0.1% sodium azide in TBS containing 0.2% Tween-20) for 30 min and then incubated with primary antibodies overnight at 4°C. Bound antibodies were detected using incubation with HRP-conjugated secondary antibodies in TBST containing 5% PVP and 5% FCS. Details of primary and secondary antibodies were listed in the Supplementary Tables 1 and 2. Images were visualized by Luminescent image analyzer LAS-4000 (Fujifilm) after adding ECL plus (GE Healthcare Life Science), and the density of each band was analyzed with the Gelscan V5.1 program (BioSciTec) [54].

### Cytochrome *c* release

DLBCL cells were spun down at 1200 × *g* for 5 min, and cytosolic protein were extracted by suspending cells into the basic buffer containing 0.02% digitonin and incubated on ice for 10 min with gentle mixing. After centrifugation at 8000 rpm for 2 min, the cytosolic extracts were collected for determining cytochrome *c* release, using Human Cytochrome *c* Quantikine ELISA Kit (R&D Systems) in accordance with the manufacturer's recommendations. In brief, cytosolic proteins (50 μg in 100 μl) were added to each well and incubated for 2 hours. After wash, cytochrome *c* in the cytosol was incubated with the conjugate solution for 2 hours, and then the substrate solution for 30 min in the dark. The absorbance values were read at 450 nm with spectrophotometry after adding the stop solution.

### Caspase activation

Cytosolic proteins (25 μg) were diluted to 90 μl with fluorogenic assay buffer. The reaction was initiated by addition of 10 μl of 400 μM (final concentration was 40 μM) fluorescent substrates: Ac-DEVE-AFC or Ac-LEHD-AFC for the activity of caspase-3 or caspase-9 (Merck-Calbiochem), respectively. The cleavage reaction was carried out at 37°C for 15 min. The fluorescence at 400/505 nm was measured with a BMG LABTECH POLARstar OPTIMA Microplate Reader [11].

### Co-immuno-precipitation (Co-IP)

Two different protein extraction methods were used for co-IP. Proteins extracted using CelLytic^™^ M cell Lysis Reagent supplied with protease inhibitor cocktails were used for detecting bindings between pan Bax and Drp1. Proteins extracted with CHAPS-containing buffer (10 mM Hepes, pH 7.4, 150 mM NaCl, 1% CHAPS, 1 mM DTT, 0.1 mM PMSF and protease inhibitor cocktails) were used for detecting bindings between active Bax and Drp1. Co-IP was performed using Dynabeads protein A (Life Technologies) [59]. Dynabeads in 50 μl of TBST were incubated with 5 μg of rabbit Bax (N-20), mouse anti-Bax (6A7), rabbitanti-DRP1 antibody, or mouse IgG, respectively, for 1 hour at room temperature on a rotator. After washing for three times with TBST, Dynabeads protein A coated with antibody or IgG were mixed with 500 μg proteins and incubated for 1 hour at room temperature on a rotator. Protein complexes were eluted by boiling with a loading buffer.

### Transfection of siRNAs

Human DRP1 siRNA and control siRNA (Santa Cruz, sc-43732; sc-37007) were used for knocking down protein expression. Cells (5 × 10^6^/ml) were suspended in 100 μl of Human B Cell Nucleofector (R) reagent (Lonza) and 2 μg of siRNA was added into the mixture. The transfection was performed using Nucleofector^™^ II apparatus with the program D-033 (Lonza). Protein expression was determined after 24 hours transfection.

### Statistical analysis

Statistical analysis was performed using GraphPad Prism version 5.03. Data are shown as mean ± SD of at least three independent experiments. Significant difference between groups with equal numbers was analyzed by two-sided Student *t* test, and groups with unequal numbers were analyzed with the *Mann*-*Whitney U*-test. Two-way ANOVA with Bonferroni post-hoc test was used to compare time-dependent changes in protein expression. Correlation between groups of variables was analyzed with Pearson's correlation. All *P*-values less than 0.05 were considered statistically significant. *P < 0.05, **P < 0.01, and ***P < 0.0001.

## SUPPLEMENTARY FIGURES AND TABLES


