# Supplementary material for: Dynamin-related protein Drp1 is required for Bax translocation to mitochondria in response to irradiation-induced apoptosis
Source: Oncotarget. 2015 Jun 4;6(26):22598–612. doi: 10.18632/oncotarget.4200 (PMC4673185; doi:10.18632/oncotarget.4200)
Supplement: Supplementary file 1 [file oncotarget-06-22598-s001.pdf]

SUPPLEMENTARY FIGURES AND TABLES

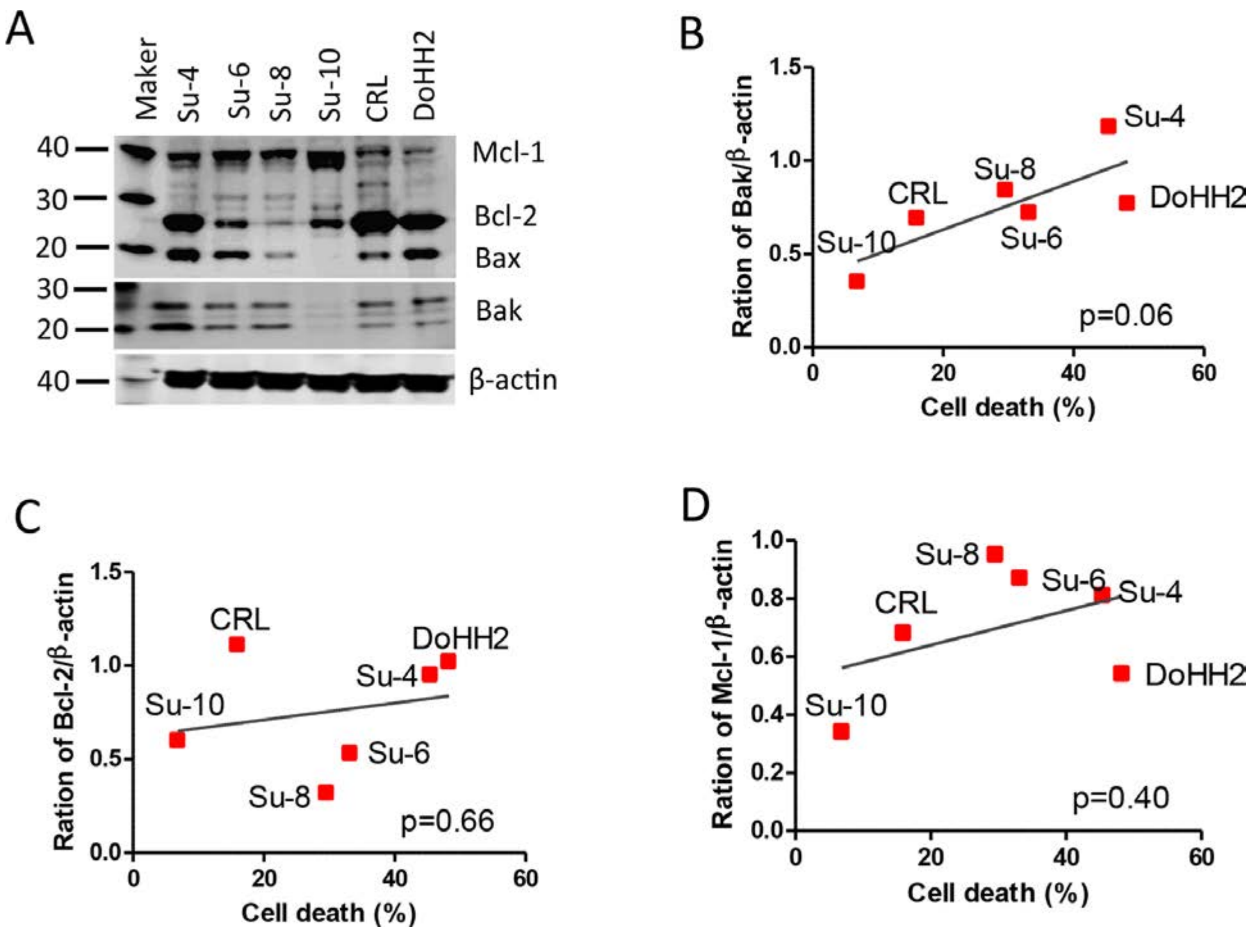

Supplementary Figure S1: Correlation between expression of Bcl-2 family proteins and the sensitivities of DLBCL cells to UV-induced cell death. A. Western blotting of Bcl-2 family of proteins. Correlation between expression of Bak **B.** Bcl-2 **C.** and Mcl-1 **D.** and the sensitivities of cells to UV-induced cell death.

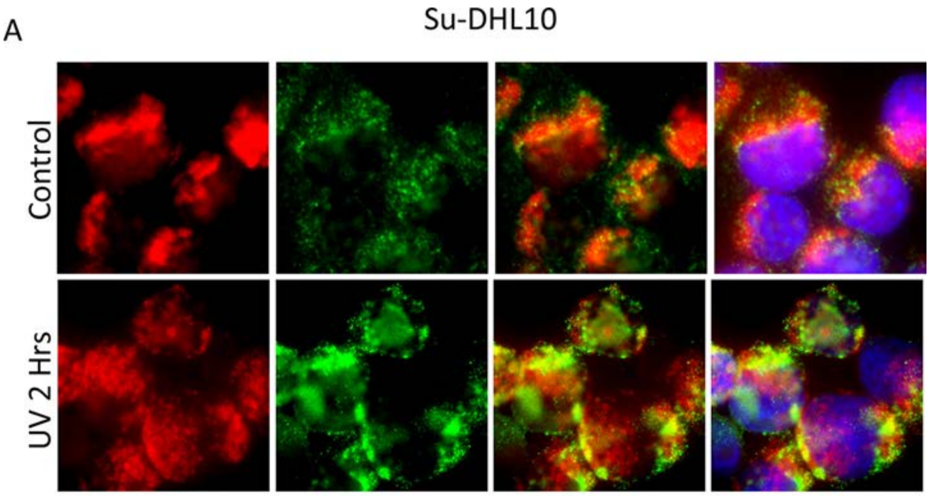

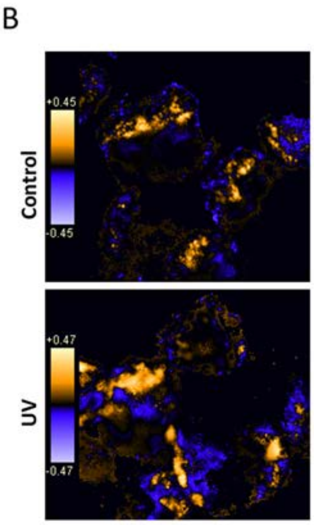

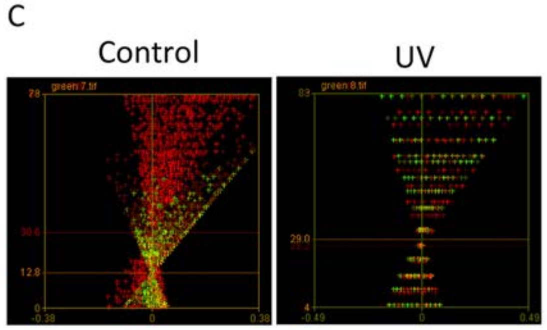

D

	Rr	R	ch1:ch2	M1	M2	N+ve
**Control**	0.339	0.792	0.872	0.879	0.807	13819
**UV**	0.438	0.844	1.732	0.787	0.999	16139

Supplementary Figure S2: Drp1 and mitochondrial colocalization analysis in Su-10 cells. A. Fluorescent staining of mitochondria (red) and Drp1 (green). **B.** PDM iamges. **C.** ICA plots. **D.** Correlation analysis.

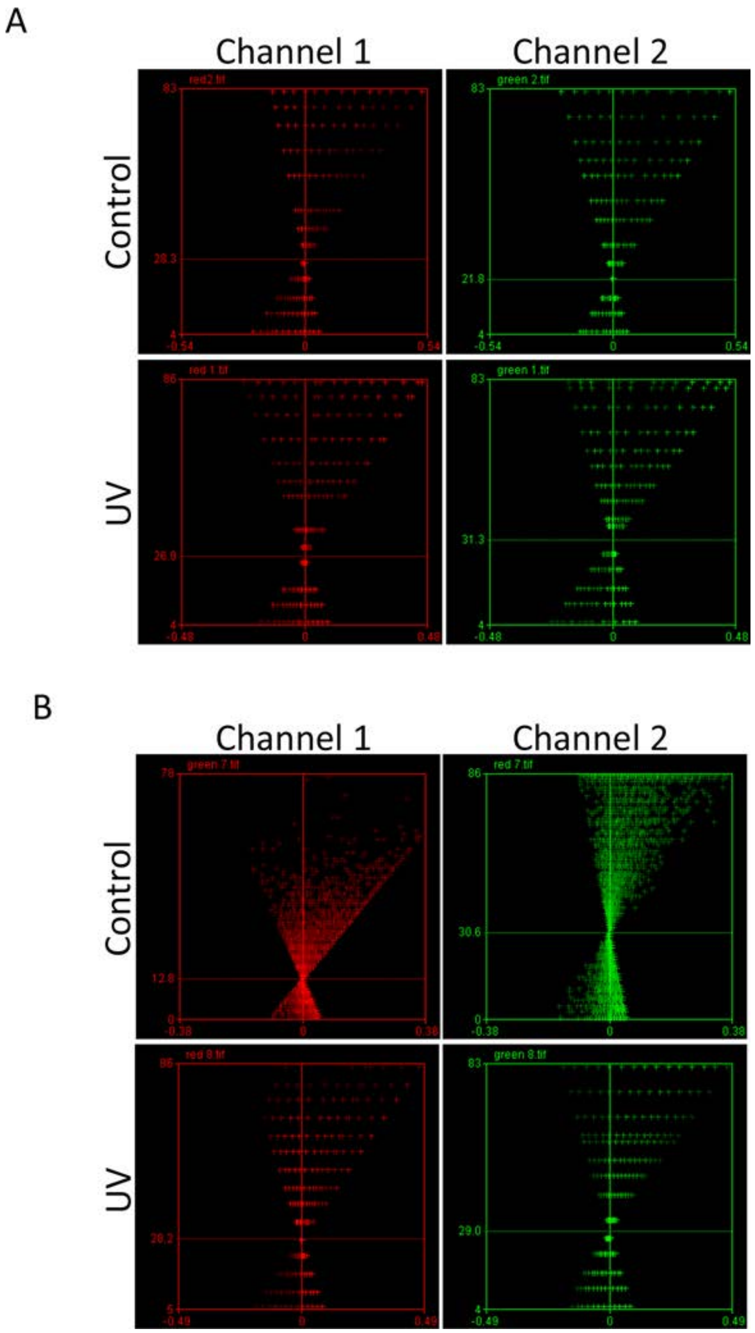

Supplementary Figure S3: ICA plots. Drp1 (green) and mitochondrial (red) colocalization in Su-4 **A.** and Su-10 **B.** Cell lines.

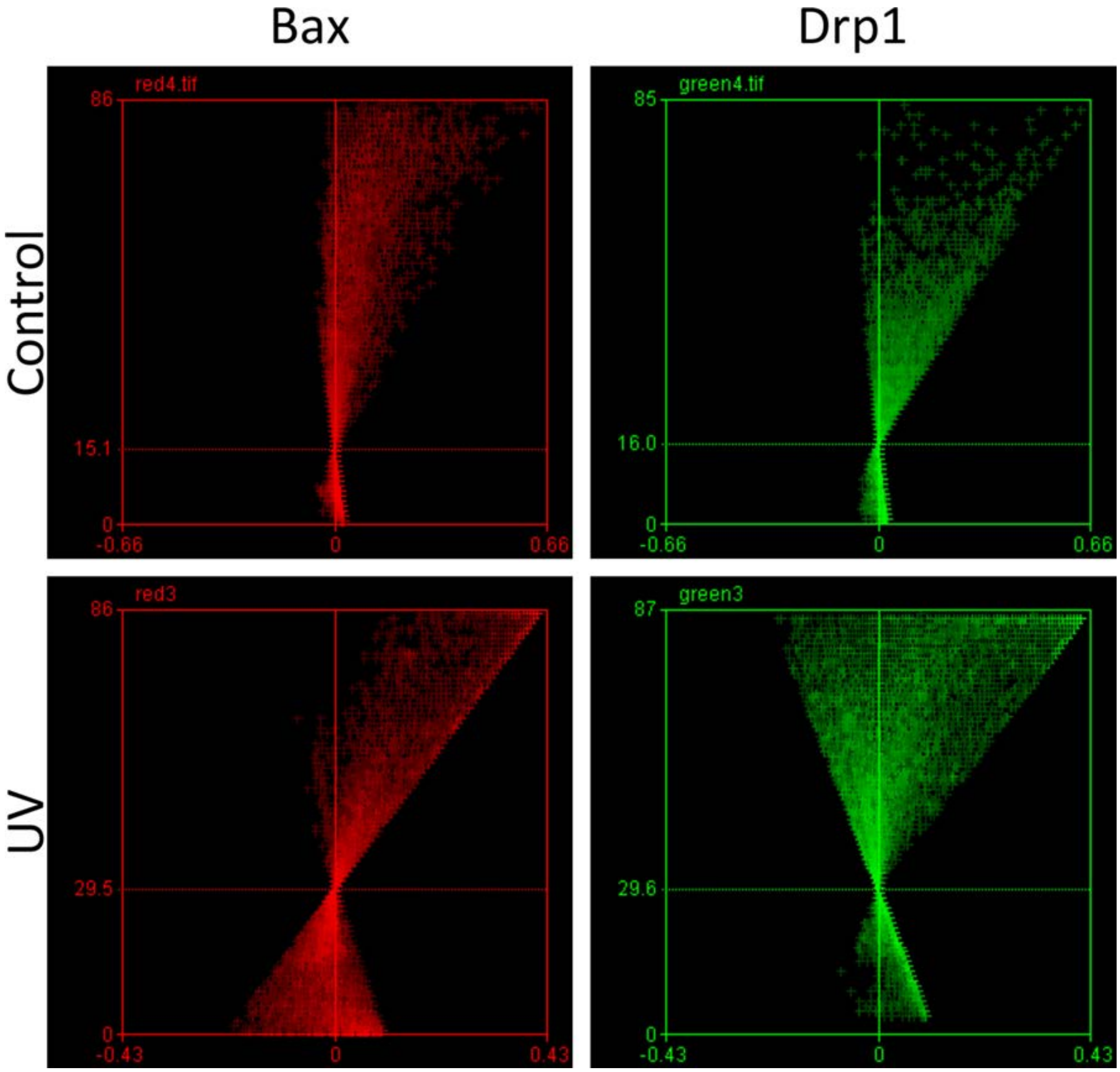

Supplementary Figure S4: ICA plots for intensities of Bax (red) and Drp1 (green).

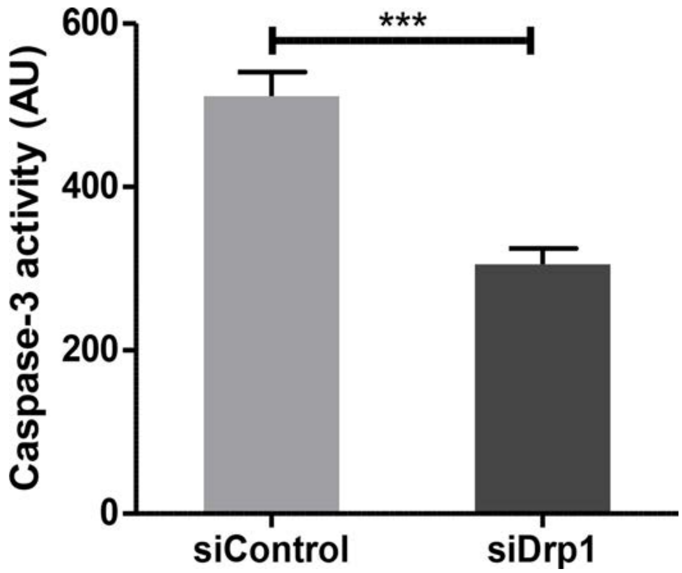

Supplementary Figure S5: UV irradiation-induced activation of caspase-3. Su-4 cells were transfected with either control siRNA (siControl) or Drp1-siRNA. After 24 hours of transfection, cells were treated with UV for 5 min and caspase-3 activity was measured after further cultured for 12 hours. Data shown were mean ± SD from three independent experiments.

Supplementary Table S1. Primary antibodies used in this study

Name of antibody	Type	Company	Cat No	Application	Dilution
**β-actin (AC-74)**	Mouse	Sigma	A5316	WB	1:250, 000
**Bax (6A7)**	Mouse	BD	556467	IF<br>IP	1:100<br>5 μg
**Bax (N20)**	Rabbit	Santa Cruz	sc-493	IF<br>WB<br>IP	1:20<br>1:500<br>5 μg
**Bax (2D2)**	Mouse	Santa Cruz	sc-20067	WB	1:1000
**Bcl-2 (100)**	Mouse	Santa Cruz	sc-509	WB	1:1000
**COX IV (G-20)**	Mouse	Santa Cruz	sc-376731	WB	1:500
**Drp1 (6Z-82)**	Mouse	Santa Cruz	sc-101270	IF	1:20
**Drp1 (H-300)**	Rabbit	Santa Cruz	sc-32898	WB<br>IP	1:200<br>5 μg
**Mcl-1 (B-6)**	Mouse	Santa Cruz	sc-74436	WB	1:200
**PARP (1/2)**	Rabbit	Santa Cruz	sc-7150	WB	1:500
**Phospho-Drp1 (Ser637)**	Rabbit	Cell Signalling	4867	WB	1:1000
**PGAM5 (K-16)**	Goat	Santa Cruz	sc-161156	WB	1:200

IF = Immuno-fluorescent staining; IP = immune-precipitation; WB = Western blotting

Supplementary Table S2. List of secondary antibodies

Name of antibody	Type	Company	Cat No.	Application	Dilution
**Anti-mouse IgG-HRP**	Goat	Santa Cruz	sc-2005	WB	1:5000
**Anti-rabbit IgG-HRP**	Goat	Santa Cruz	sc-2004	WB	1:5000
**Anti-goat IgG-HRP**	Donkey	Santa Cruz	sc-2020	WB	1:5000
**Alexa Fluor® 546 Anti-rabbit IgG**	Goat	Invitrogen	A11035	IF, FC	1:100
**Alexa Fluor® 488 Anti-Mouse IgG**	Donkey	Invitrogen	A21202	IF, FC	1:100
